# Leveraging Text Messaging and Mobile Technology to Support Pediatric Obesity-Related Behavior Change: A Qualitative Study Using Parent Focus Groups and Interviews

**DOI:** 10.2196/jmir.2780

**Published:** 2013-12-06

**Authors:** Mona Sharifi, Eileen M Dryden, Christine M Horan, Sarah Price, Richard Marshall, Karen Hacker, Jonathan A Finkelstein, Elsie M Taveras

**Affiliations:** ^1^Division of General PediatricsMassachusetts General Hospital for ChildrenHarvard Medical SchoolBoston, MAUnited States; ^2^Institute for Community HealthCambridge Health AllianceHarvard Medical SchoolCambridge, MAUnited States; ^3^Department of Population MedicineHarvard Pilgrim Health Care Institute and Harvard Medical SchoolBoston, MAUnited States; ^4^Harvard Vanguard Medical AssociatesBoston, MAUnited States; ^5^Division of General PediatricsBoston Children's HospitalHarvard Medical SchoolBoston, MAUnited States

**Keywords:** child, obesity, overweight, health behavior, text messaging, telemedicine

## Abstract

**Background:**

Text messaging (short message service, SMS) is a widely accessible and potentially cost-effective medium for encouraging behavior change. Few studies have examined text messaging interventions to influence child health behaviors or explored parental perceptions of mobile technologies to support behavior change among children.

**Objective:**

Our aim was to examine parental acceptability and preferences for text messaging to support pediatric obesity-related behavior change.

**Methods:**

We conducted focus groups and follow-up interviews with parents of overweight and obese children, aged 6-12 years, seen for “well-child” care in eastern Massachusetts. A professional moderator used a semistructured discussion guide and sample text messages to catalyze group discussions. Seven participants then received 3 weeks of text messages before a follow-up one-on-one telephone interview. All focus groups and interviews were recorded and transcribed verbatim. Using a framework analysis approach, we systematically coded and analyzed group and interview data to identify salient and convergent themes.

**Results:**

We reached thematic saturation after five focus groups and seven follow-up interviews with a total of 31 parents of diverse race/ethnicity and education levels. Parents were generally enthusiastic about receiving text messages to support healthy behaviors for their children and preferred them to paper or email communication because they are brief and difficult to ignore. Participants anticipated high responsiveness to messaging endorsed by their child’s doctor and indicated they would appreciate messages 2-3 times/week or more as long as content remains relevant. Suggestions for maintaining message relevance included providing specific strategies for implementation and personalizing information. Most felt the negative features of text messaging (eg, limited message size) could be overcome by providing links within messages to other media including email or websites.

**Conclusions:**

Text messaging is a promising medium for supporting pediatric obesity-related behavior change. Parent perspectives could assist in the design of text-based interventions.

**Trial Registration:**

Clinicaltrials.gov NCT01565161; http://clinicaltrials.gov/show/NCT01565161 (Archived by WebCite at http://www.webcitation.org/6LSaqFyPP).

## Introduction

The public health significance of childhood obesity is well-known, as are the complexities involved in supporting obesity-related behavior change in clinical practice. The ubiquitous use of mobile phones in the United States [1] may provide opportunities to support families in health behavior change through the innovative use of applications such as short message service (SMS) text messaging. Text messaging is a widely accessible and potentially cost-effective medium for facilitating behavior change through support and immediate feedback capabilities. In 2012, over 2.3 trillion text messages were sent, and there were more active cell phones than people in the United States [2]. Rates of mobile phone use are highest among historically difficult to access populations including adolescents, young adults, low-income populations, less educated adults, and those with less stable home addresses [3-6].

Prior studies have assessed the influence of text messaging on adult weight loss [7-9], smoking cessation [10,11], asthma management [12], sunscreen application [13], heart failure self-management [14], prenatal care [15], and medication adherence [16]. Fewer studies have been conducted among children and/or their parents. For example, text messaging to support weight management was found to be feasible and acceptable among adolescents [17,18] and has been used to promote adolescent sexual health [19], physical activity [20], and diabetes self-management [3,21,22]. Text messaging directed to adolescents and parents was found to reduce “no-show” rates to medical appointments [23] and promote adherence to vaccination schedules [24,25]. These studies show promising results, yet few studies have assessed text messaging to parents to support ongoing and complex health behaviors such as overweight and obesity management for their children. Parents play a key role in helping their children navigate obesogenic environmental and media influences and as agents of change in modifying weight-related behaviors [26]. Further information is needed to facilitate the design of mobile technology-powered pediatric obesity interventions that target parents as agents of change.

The purpose of this study was to explore parental acceptability and preferences regarding the use of text messaging and other mobile technologies to support pediatric obesity-related behavior change.

## Methods

### Study Setting and Population

We conducted five focus groups and seven follow-up interviews with parents of overweight or obese children, aged 6-12 years, receiving care at one of two pediatric practices in eastern Massachusetts. We purposively selected two pediatric practices for recruitment in order to obtain heterogeneity in the sociodemographic characteristics of participants. One practice was a part of Cambridge Health Alliance (CHA), a safety net institution [27] caring for an ethnically diverse and traditionally underserved population. The other was part of Atrius Health, a private institution serving families with a broad range of socioeconomic backgrounds.

Focus group eligibility included parents who (1) had a child 6.0-12.9 years old with a body mass index (BMI) ≥85^th^ percentile for age and gender at the most recent “well-child” visit with no other chronic conditions, (2) could communicate in English, and (3) had a cell phone capable of text messaging. The study focused on parents of overweight and obese children in order to obtain opinions and preferences relevant to this high-risk population. Further, the study was limited to school-age children rather than younger children or adolescents, who are distinct in their levels of autonomy over their behaviors and environments.

### Recruitment and Enrollment

Participants were recruited from a sample of parents with children meeting the eligibility criteria who had a well-child visit at CHA’s Somerville Pediatrics practice between July 2010 and August 2011 or at Atrius Health’s Dedham Medical Associates (DMA) between April 2011 and September 2011. Study staff sent recruitment letters, with an opt-out phone number, to the parents of 388 children seen at CHA and 297 children seen at DMA. Two parents from CHA and three from DMA called to opt-out. Seven days after mailing the letter, two research assistants began recruitment calls starting from the top and bottom of an alphabetical list of remaining children to establish eligibility, explain the study, answer questions, and schedule parents for focus groups. After attempting calls to over 60% of the CHA sample and the entire DMA sample, they scheduled 39 participants for three focus groups at CHA and 30 parents for two groups at DMA. Calls were discontinued once 12-15 participants were recruited for each group.

During focus groups, the moderator invited parents to submit their contact information voluntarily for potential inclusion in a 3-week mock text messaging intervention and follow-up interview. Participants were selected through stratified random sampling from among volunteers to ensure that 1-2 participants from each focus group were included in interviews. From 11 volunteers, 8 parents were selected and 7 completed interviews.

### Data Collection

The study team, which included pediatricians, health services and public health researchers, and an anthropologist, created the focus group guide informed by standard focus group techniques [28] and existing literature on text messaging and mobile technologies. Through several iterations, the focus group guide ultimately included 26 open-ended core questions derived from the study aims and research questions. These questions were supplemented by spontaneous probes and follow-up questions during the focus group discussions. Prior to starting the focus groups, participants completed a brief, anonymous survey on demographic characteristics and mobile technology use and preferences. The focus group questions addressed parents’ perspectives regarding the use of mobile technologies to support them in helping their children adhere to evidence-based behavior goals for obesity prevention (eg, reducing sugar-sweetened beverages and fast food, increasing physical activity, ensuring adequate sleep, and limiting screen time) [29]. Discussion questions focused on message design, clarity, content, relevance, personalization, and potential effectiveness in supporting behavior change. In order to catalyze discussion during focus groups, parents received six sample text messages about health behaviors on their own mobile phones, including tips and self-monitoring messages to which they were encouraged to reply via text in real time (Textbox 1). In one focus group, text messaging failed and participants received all messages on paper. The focus groups lasted between 90 and 120 minutes, and participants received a light meal and a $30 gift card for their time.

**Sample text messages received by participants during focus groups and before follow-up interviews.**

Sample text messages received by participants during the focus groups

- Most juices and sports drinks are loaded with sugar and calories -- even 100% juice is. When your child is thirsty, water should be the drink of choice.
- How many hours or minutes did your child watch or use screen media on an average day in the last week? *Please text back your response* Thanks!
- Responses (*reviewed on paper*)

      • If meeting goal: Keep it up! You’re off to a great start.

      • If almost there: That’s not far off the recommended amount. The goal for this behavior is within reach!

      • If needs work: There is work to be done to reach the recommended amount for this goal, but your child can do it. Start with small changes and build up.

- Your child’s doctor suggests less than 2 hours a day of screentime. This is time on TV, DVDs, movies, computer & video games. Handheld devices count too!
- Well-rested children make for happier parents. 6-12 year-olds need 10-11 hrs of sleep, and enough sleep can make for better moods and better learning.
- Run, walk, ride, skate, dance, play. What exercise does your child enjoy? The goal is 1 hr/day. If it sounds like a lot, start slowly and build up.

 

Sample text messages received by participants during the 3-week mock intervention

Week 1:

- Work on the 10-2-1-0 goal for kids! That’s 10 hours of sleep, less than 2 hours of TV, 1 hour of physical activity, and 0 sugary drinks.
- How many hours did your child sleep in an average night in the last week? *Please text back your response* Thanks!
- Self monitoring response

      Examples:

      • Great job! Congratulate your child for being on target with this behavior.

      • Your child is close to meeting the recommendation. Work together to think of small steps to help them reach the goal.

      • There’s a ways to go to meet the goal, but work with your child to start slowly and keep at it.

Week 2:

- Just say no to TV in kids bedrms. Studies show that your child may be more willing to go to sleep, fall asleep faster, & sleep longer w/o TV in the bedrm.
- Skip sugary drinks for kids. Choose water instead. Seem too plain? One parent told us she makes it fun with orange or lemon slices or sparkling water.
- Kids need 1+ hrs moderate-vigorous activity a day. What’s that? If moderate, you can talk, not sing. If vigorous, you can say 3 words, then need a breath.

Week 3:

- How many hours or minutes did your child do moderate to vigorous physical activity on an average day in the last wk? *Please text back your response* Thanks!
- Self monitoring response (see examples above)
- Remember, you are your child’s role model! Limit your own TV time to help your children do the same.

Focus group volunteers chosen to participate in the mock text messaging intervention received three text messages a week for 3 weeks (Textbox 1) before a one-on-one telephone interview approximately 3-4 weeks after the focus group. In order to deliver the text messages, we contracted with Mobile Commons (Brooklyn, NY), a mobile technology vendor that offers a Web-based platform to support text messaging programs. The follow-up interview guide was informed by findings from the focus groups and further explored parents’ real-life experience receiving text messages. Interviews lasted about 30 minutes, and participants received a $30 gift card for their time.

All participants gave written consent to participate in focus groups and interviews. The Harvard Pilgrim Health Care Institute Human Studies Committee and the CHA Institutional Review Board for Human Subjects Protection approved this study.

### Analysis

We used descriptive statistics to summarize participant characteristics based on survey data. The focus groups and interviews were audio-recorded, transcribed verbatim, and imported into QSR International’s NVivo 9 software, a qualitative data analysis program. Using a framework analysis approach [30], 2 members of the research team (MS and ED) developed an initial codebook of themes that included a priori themes of interest and new themes that arose after both researchers attended all focus groups and read all transcripts in their entirety. Three research team members (including a qualitative research expert, ED) then coded two transcripts independently and met to reach consensus about the codes to be used. Additional codes were then included in the codebook as new themes emerged. The same team members coded the remaining focus group and interview transcripts while meeting periodically to review and discuss any discrepancies in coding. Analysis involved the systematic comparison of coded segments across the five focus groups and seven interviews transcripts to identify convergent, salient, and/or unique themes. Research staff compiled and shared these interpretations with the larger research team for their final review.

## Results

### Participant Characteristics and Mobile Technology Use and Preferences

We completed five focus groups with 31 parents (28 mothers and 3 fathers) and seven follow-up interviews with focus group participants of diverse race/ethnicity and education levels. Table 1 shows results from the brief survey of participants on demographic characteristics as well as mobile technology use and preferences.

The majority of participants had unlimited text messaging cell phone plans (77%, 24/31) and reported text messaging at least once a day (71%, 22/31). A small minority reported any mobile service interruptions (10%, 3/31). Eleven participants (35%, 11/31) reported enjoying texting “a lot” with the remainder reporting “somewhat” (48%, 15/31) or “a little” (13%, 4/31). Only one parent reported ever having signed up to receive text messages about health information. Barriers to more frequent texting listed by parents included cost, preference for talking on the phone, and lack of interest. Among the 19 smartphone owners in the group, 16 reported ever having downloaded and used apps (applications) on their smartphone, and only 2 had ever downloaded a health-related app such as a weight or fitness tracker. All 31 parents reported that they would like to receive text messages from their pediatrician’s office with advice about their children’s health, and all but one parent were willing to reply via text regarding their children’s health behaviors.

### Text Messaging Acceptability

Table 2 presents the major themes and representative quotes that emerged from the focus groups regarding text messaging acceptability. Parents were enthusiastic about text messaging interventions and most commonly cited convenience and “ease of use” as advantages. Many characterized text messaging as more effective than other types of communication because its brevity, immediacy, and “hard to ignore” quality make parents more likely to read messages. While some reported more comfort with email than text messaging, many felt inundated with emails and reported consequently ignoring or overlooking many. Some considered the asynchronous nature of text messaging a benefit because you can read and reply to messages when convenient. Many mentioned that text messages are easy to share and show to others, allowing quick dissemination of health messages to family and friends.

Limitations of text messaging described by parents included the difficulty of referring back to messages at a later time and message size constraints. A few parents described less comfort with the technology, and some felt cost would be a barrier for those without unlimited text messaging plans.

**Table 1:** Participant characteristics and mobile technology use and preferences among 31 participants in parent focus groups.

Characteristics			Mean (SD) or n (%)
**Participant characteristics**			
	Parent age, yrs (range 28-51)		41.1 (6.3)
	Child age, yrs		8.7 (1.9)
	**Race/Ethnicity**		
		White	20 (65%)
		Black	3 (10%)
		Hispanic	3 (10%)
		Other	5 (16%)
	English primary language		20 (65%)
	**Education completed**		
		High school	10 (32%)
		College	11 (35%)
		Post-graduate	8 (26%)
**Mobile technology use & preferences**			
	Text once per day or more		22 (71%)
	Enjoy texting “a lot” or “somewhat”		26 (84%)
	Unlimited text messaging plan		24 (77%)
	Ever signed up for texts about health information		1 (3%)
	Own a smartphone that can connect to the Internet		19 (61%)
	Ever downloaded an app		16 (52%)
	Ever downloaded a health-related app		2 (6%)
	**Mobile service interruptions (last 12 months)**		
		Never	27 (87%)
		1-2 times	3 (10%)
	**How frequently would you like to get text messages from your pediatrician’s office with tips/advice about your child’s health?**		
		Daily	1 (3%)
		4-6 times per week	2 (6%)
		1-3 times per week	9 (29%)
		1-3 times per month	17 (55%)
		Less than once per month	2 (6%)
		Never	0 (0%)
	Willing to reply to text messages from your pediatrician’s office about your child’s health behaviors?		30 (97%)

**Table 2:** Perceptions of text messaging to parents to support child behavior change.

Perceptions		Representative quotations
**Advantages of text messaging**		
	Convenient	“I don’t have to try to find the information. It comes to me.”
		“I’m always on my phone. It’s just easier.”
		“You get a better chance of me getting the message immediately if it’s a text...I just have to read so many emails.”
	Brief	“You text people because you don’t want to pick up the phone and get into a half an hour conversation. You want it to be brief.”
		“I think the pro is that text is brief and it’s done and it’s over with”
	Hard to ignore	“It’s immediate, and there’s something about a text that makes you just have to look at it.”
		“pay more attention…read it right away”
	Asynchronous	“My favorite form of communication is the text message just because…you don’t have to answer it immediately.”
	Reminder	“I already know that but it’s kind of like…the little angel and devil on your shoulder.”
	Easy to Share	“I wouldn’t mind them seeing some of this so it’s not constantly, ‘Mom says I can’t do this, mom says I can’t do this.’”
**Limitations of text messaging**		
	Space limits	“harder to consume a lot of information”
	Variable comfort	“I didn’t start to text until the last year or two.”
		“If she had texted me, I wouldn’t have been able to respond.”
	Hard to reference	“If I go and delete that text message, I can’t get it back.”
	Cost	“They should know who has the [unlimited text messaging] plan… like not presuming everybody has [it].”

### Text Messaging Preferences and Intervention Recommendations

#### Overview

Table 3 shows participants’ preferences and recommendations for text messaging interventions to support healthy behaviors for children summarized in the following categories.

#### Content

Parents expressed a need for specific, action-oriented advice and strategies to achieve goals rather than general information on healthy behaviors. Examples of useful information included alternatives to screen time, a “do this, not that” list (eg, a list comparing the effectiveness of a variety of physical activities), lists of healthy snacks, tips/recipes for healthy yet inexpensive meals, and local physical activity events/programs. Initially, some felt they would appreciate messages only about behaviors needing improvement; yet, the sample text messages about behaviors in which their children were excelling were found to be “encouraging”. These parents ultimately stated they would prefer a mix of messages about behaviors their child was doing well and ones that needed work. Participants recommended keeping messages focused on one goal (or set of related goals) at a time to avoid making parents feel overwhelmed with numerous health behaviors; some suggested having weekly themes for message topics. Several parents expressed concerns about creating body image issues and disordered eating habits among their children and wanted sensitive and non-stigmatizing strategies to prevent these issues.

#### Frequency/Timing/Duration

After receiving the sample text messages, the majority of parents felt that receiving messages twice per week would be appropriate; some parents said they preferred daily messages. This was a notable shift in preference toward more frequent messages compared to the results of the survey completed prior to beginning the groups. There was no consensus on the best time of day to receive messages or even on unacceptable times. Participants felt strongly that they could continue to receive messages indefinitely if the content remained relevant and novel. There was consensus that there should be an option to increase or decrease the frequency of messages.

#### Voice of Authority

Participants frequently noted that a text from a health care provider would have a “voice of authority”. They anticipated high responsiveness from children simply because the source is someone other than the parent and felt that showing a child or other caregiver a text message could serve as proof to validate the parents’ efforts to encourage behavior change.

#### Personalization/Customization

Parents felt that the more relevant messages are to their child, the more effective they will be in supporting behavior change. Suggestions for creating and maintaining relevance included tailoring messages to the child’s age, gender, neighborhood, health conditions, preferred goals, and current knowledge. Some imagined messages with the child’s name and even their doctor’s name to make them personal and direct.

**Table 3:** Parent preferences for text messaging interventions to support child behavior change.

Topic area		Representative quotations
**Content**		
	Specific strategies, “how-to” advice, and resources	“I know what’s good to do [but] how do I get my son to do things I want him to do.”
		“Everything I get is very generic...‘Let’s eat more vegetables, and let’s not go to McDonald’s’, which doesn’t actually apply. So it’s not necessarily directives or ideas or strategies”. “‘Your kid should exercise.’ Really? I know that. Or, like, ‘Drink water.’ Come on, we know that. Give me something interesting, like there was a new study out, and your kids should eat more of this.”
	Weekly themes targeting one health behavior	“I like the idea of themes...If I had to deal with food and screen time and bed time all in the same week, we’d all end up in the nuthouse.”
		“If it’s random stuff all week, I mean, every week, I would probably be like, ‘OK, enough,’ you know?”
	How to prevent issues with body image and disordered eating	“I’m really nervous on how to talk to her…because I don’t want her to feel like there is anything wrong with her… figuring out the best way to approach it so she’s OK with herself and her body image while embracing the correct behaviors to make sure she stays healthy.”
		“...how would I approach my daughter about food because I don’t want her to keep worrying about ‘I have to eat this, I’m not supposed to eat that.’ I don’t want to stress her out about that.”
		“How do you make this not something about shame and how do you make it something about healthy eating habits? “…to say, ‘Well, you’re a little overweight,’ that would really crush him. You have to be very careful”
**Duration and frequency**		
	Contingent on relevance	“If it follows your child as your child ages, it could go on until the child is an adult…there could always be new things to say.”
		“If you are getting these once a week and now its six weeks later and you haven’t really gotten any information that’s interesting to you, then I think I would text stop.”
		“It starts repeating, you’d be like, ‘OK, I’ve heard it all’.”
	Authoritative source	“I think some kids will listen to their doctor better than their parents”
		“Neither my son nor my husband listen to anything I have to say unless, like, I show them on a website.”
		“It’s not like your parents are telling you because, of course, whatever your parents say, it’s obviously never true…”
	Personalized	“Choose your own adventure”
		“Here’s a great thing you can do with an avocado…and they’re on sale at [the nearby] Market Basket.”
		“Have the option to change because sometimes one thing is working real well and then ... you might need more information on another topic.”
		“What if you filled like a little survey and...I get to pick [the topics]. Don’t send me information about this…Every once in a while you can throw some in, but I really want to focus on these.”
	Interactive	“I would love to ideally be able to have someone on-call on text…whatever your question is and then there’s someone that it pops up and they can text you right back.”
		“If you wanted to get more information that you could reach out. So if at the end of one of the texts it said have questions, call.”
		“It should go back somehow to the pediatrician, some way, so that we’re almost held accountable during the visit.”
		“I think if it becomes a mechanism for you to have some kind of ongoing dialogue—a way that you feel connected to your primary care physician, then I think that would extend the longevity of how long I would stay invested in the program.”
	Multimodal	“You could text back ‘more’ and maybe something automatically shoots off to your email. You could say ‘to see more, go to this website.’ You can get that within 160 characters.”
		“If you had it all go to the same website, you could have a little blog. Somebody could say, ‘hey, do you have a healthy Halloween snack?’ And then somebody could tap in if they want to answer back.”
		“I think if you’re collecting data, there’s some kind of dashboard you see where…you have a graph that shows you your screen time use at the same time show you everybody else’s… I think that could help a lot in terms of helping people change.”
		“A supportive website that had these exact texts with an area for more resources if you wanted…I don’t need a person. Just a place to go for more information would be fine.”

#### Interactivity

Most parents preferred an option for “live” support to answer questions. While some envisioned telephone support, most indicated that responses to questions via text message would be sufficient. Some participants preferred that their pediatrician receive their responses and eventually discuss progress with them.

#### Multimodal

Parents suggested supplementing text messages by linking to email, websites, mobile apps, or patient health portals. Most parents wanted email in conjunction with texts, reasoning that email is widely available, can contain a large amount of information, and may be easier to use for those newer to text messaging. Participants felt that the use of mobile phone apps would be attractive to younger parents. We observed that the discussion around mobile apps was more active among parents at DMA than CHA with an apparent higher acceptability for mobile apps and mobile interventions directly targeted to their children among DMA parents.

Parents frequently discussed integration of text messaging with a website that could record and track progress, perhaps in relation to how others are doing, and serve as a “one-stop shop” for supportive advice, information on local resources and activities, and access to forums, support groups, and research findings. Some reported positive experiences using patient health portals, and one parent recommended tracking parent responses to text messages within their child’s patient portal to facilitate communication with the doctor.

### Experiencing the Mock Text Messaging Intervention

The focus groups included rich discussions about hypothetical text messaging interventions. Follow-up interviews with 7 focus group participants who experienced a 3-week mock text messaging intervention (Textbox 1) substantiated most of the themes generated during the focus groups. These participants felt strongly that text messaging is a convenient way to receive health messages and reiterated the benefits of the immediacy and asynchronous nature of texts. While very little of the information was new to them, they found the majority of information received to be helpful reminders of positive health goals. One parent reported that she “liked the attention [from] people interested in how we manage it with the kids.” Receiving three messages a week between the times of 8 a.m. and 5 p.m. seemed appropriate to most. Two participants would have liked to receive even more messages, while one said they were “kind of a little too many” and stopped responding to messages because she found it burdensome to do so during the holiday season. Many suggestions mirrored those from focus groups: more specific suggestions, personalization, access to a “real person” for questions, and integration with email and Web to enhance interactivity and provide access to more information.

Some parents reported behavior change among their children during the 3-week mock intervention. The ability to share messages, which the children perceived to be from their doctor, was felt to be quite powerful. One parent said, “Every time I tell her she doesn’t listen to me. I said this came from your doctor’s office, and she was happy and said, now I have to start drinking water.” Two others reported that their children started going to bed earlier, drinking more water, and exercising more. Another participant related a conversation with her son prompted by a text message about the difference between moderate and vigorous exercise. The result of the conversation was that her son chose to go running that afternoon with her because it was more vigorous.

## Discussion

### Principal Findings

In this qualitative study, parents of overweight and obese children aged 6-12 years reported general acceptability and enthusiasm for text messaging interventions supporting healthy behaviors for children. Parents found text messaging innovative and preferable to paper or email communication because it is immediate, brief, and difficult to ignore. Keys for creating and maintaining relevance included providing novel, relevant information with specific strategies for implementation and personalization of information specific to the child and local community. Most parents favored multimodal interventions utilizing text messaging to relay information and as a trigger or link to other forms of media including email and Web-based platforms.

Prior studies have demonstrated the promise of text messaging interventions in promoting behavior change [3,6-25,32,33], and interventions with features such as tailoring/personalization and a two-way interaction to reduce attrition have observed better results. Yet, few studies have explored the use of mobile technologies directed at parents to support complex, ongoing healthy behaviors in children. In one of the few published studies involving 31 parents and children, investigators observed greater adherence to self-monitoring of sugar-sweetened beverage consumption, physical activity, and screen time among those receiving text messages compared to those keeping paper diaries [34]. Although the study did not evaluate actual behavior or acceptability and preferences among participants, it supports the potential of text messaging as a tool for communication and health behavior tracking.

This study represents a novel exploration of parent preferences regarding text messaging and other technologies to support obesity-related behavior change for their children and presents themes that can guide future interventions. The implementation of such interventions leveraging mobile technologies presents unique opportunities as well as some challenges. Although the ubiquity of cellular telephones is increasing and bridging socioeconomic divides in access, any intervention utilizing mobile technologies like text messaging requires consistent telephone service, reliable and up-to-date contact information, and participant consent to receive text messages per Federal Communications Commission regulations [35]. Furthermore, the interest we observed among parents to receive tailored messages with options for interactivity and eventual feedback from their children’s physicians requires appropriate clinical information systems and demands policies to protect patient confidentiality. The resource implications and incremental benefit of these factors merit further evaluation.

### Strengths and Limitations

Strengths of the study design included the sample texts provided during focus groups as well as the mock intervention and follow-up interviews. A few limitations should be considered. The qualitative design and sample size are not intended to determine exact percentages of parents holding a given belief. However, themes recurred in multiple groups and interviews supporting their salience. The results may not be broadly generalizable, although participants were purposively sampled from two practices serving diverse populations. Similar themes emerged at both sites, although participants at DMA indicated higher acceptability for mobile apps and mobile interventions directly targeted to their children. Given the risk for social desirability bias wherein participants seek to appease investigators and/or conform to dominant opinions, the moderator guides were developed to facilitate frank and open discussions. Finally, information is lacking about parents who did not participate, thus response bias is also possible, that is, participants may not hold representative views regarding mobile communication technologies to support healthy behaviors.

### Conclusions

Text messaging is a promising and low-cost medium for supporting pediatric obesity-related behavior change that is easily scalable within a health care system and appears to have high acceptability among parents of overweight and obese children 6-12 years old. Studying actual behavior change in response to text messaging will be a critical next step.

## Abbreviations

BMI: body mass index
CHA: Cambridge Health Alliance
DMA: Dedham Medical Associates
SMS: short message service
